# The Silver Linings of COVID-19

**DOI:** 10.1177/2633559X211014602

**Published:** 2021-07

**Authors:** Linda Gutierrez

**Figure:**
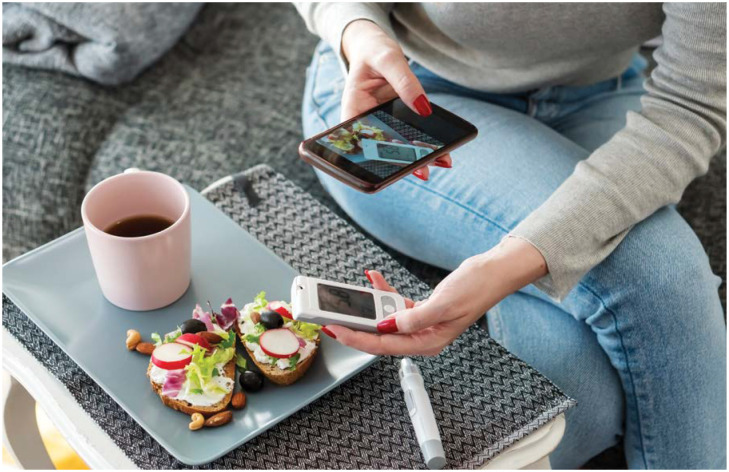


On a snowy winter day in Ohio, where my car is buried in snow, I am reminded of a silver lining of the COVID-19 pandemic—being able to help patients with diabetes from home with a telephone call. My patients and I do not have to drive to the clinic for them to receive care. As a pharmacist and certified diabetes care and education specialist (CDCES) with a consult agreement that authorizes me to initiate, adjust, and discontinue medication therapy, I can help patients reach their A1C goals over the phone. I can provide education about how medications work, go over signs and symptoms of diabetes, explain long-term consequences of diabetes, talk about nutrition, and help those with diabetes get through sick days.

Prior to COVID-19, I had not taken full advantage of telephone visits. I had also not conducted any teleheath visits before. Telehealth visits have helped me provide education through showing patients how to use injectable medication. Radio broadcasting services enabled me to inform our Spanish-speaking population of services offered at my health center. Other silver linings during the pandemic include using Zoom presentations for the first time for education, making it easier for patients to get A1C labs, and using telemonitoring and registry data to help out individuals with diabetes.

## Reaching Out to More People

I work at a federally qualified heath center in southwestern Ohio, where finding transportation to medical appointments is a concern for many people. In March 2020, our clinic moved from primarily seeing people in clinic to offering telehealth and telephone visits. For those without cars, telehealth and telephone visits have made it more convenient to receive care. For people without smartphones or computers, I can still provide care and education with a telephone call. In fact, telephone visits have let me reach out to more people than I had previously when I was doing in-person clinic appointments.

According to the Pew Research Center, those less likely to have Internet connection at home include the elderly, racial minorities, rural residents, and those with lower income or education. I have been able to provide diabetes education and help manage medications for these populations with a telephone call. I remember in particular helping out an elderly paraplegic person with diabetes who relied on the bus system for transportation. Every week for a few months, I would check in on him by phone and help him titrate his insulin. During the pandemic, there was a period where the bus system was shut down and this individual was confined to his apartment. Having weekly telephone visits allowed me to closely follow up and help him manage his diabetes.

Although telephone calls are a great way for communicating, sometimes we need to see something to learn about it. Telehealth visits proved better for showing an individual how to use insulin or a blood glucose meter. Through teleheath visits, I could observe injection technique and offer suggestions. I could also see inside the refrigerator or food pantry to help with nutritional recommendations. Teleheath visits offer another way to provide care to more people.

For our Spanish-speaking population, I appeared on a local Spanish radio station informing them about available teleheath and telephone visits. In addition to speaking about primary care, clinical pharmacy, and reproductive health services, I also emphasized the availability of comprehensive services for diabetes. Where language can be a barrier in health care, I am thankful to be able to communicate with our Spanish-speaking community as a bilingual DCES. Radio is an effective way to reach out to many people.

Telephone visits have let me reach out to more people than I had previously when I was doing in-person clinic appointments.

Like many other DCESs, I learned how to use Zoom to provide diabetes education. For the first time, I educated people in multiple states at once. I participated in a healthy cooking demonstration where I discussed nutrition while another presenter cooked a vegetable medley. I also talked about the National Diabetes Prevention Program and what it means to live with diabetes. Another time, I initially was scheduled to give an in-person presentation about diabetes costs and prevention at an occupational safety meeting, but due to COVID-19, it turned into a Zoom presentation where others outside of Ohio could also attend.

## Curbside A1C

Due to COVID-19, our clinic implemented curbside A1C labs to allow people to get the test done in their car. This helped reduce exposure risks by avoiding going into the clinic. Previously, A1C tests were done during in-clinic provider visits. Having the option of a curbside A1C has made getting the test more convenient. This has made it easier to follow American Diabetes Association guidelines to obtain AIC measures every 3 to 6 months. Many now get a curbside A1C before a scheduled telehealth or telephone visit.

Due to COVID-19, our clinic implemented curbside hemoglobin A1C labs to allow people to get the test done in their car.

## Registry Data

As a result of not being in clinic, where I could personally meet people with diabetes, I needed to find a way to identify those who needed diabetes education or medication management. For the first time, I took advantage of using registry reports that identified individuals with diabetes via their last A1C. I reached out to those who were due or past due for A1Cs and scheduled curbside A1C tests as well as appointments with a primary care provider and a DCES. The registry reports also allowed me to focus on those with very high A1Cs, who might need more diabetes education.

## Telemonitoring

During the pandemic, I have utilized the benefits of telemonitoring. I signed people up for free blood glucose telemonitoring services offered by a Medicaid managed care organization in Ohio in which self-monitored blood glucose values are reported to the clinic. Individuals and their providers are called if hypoglycemia or hyperglycemia is noted based on glucose parameters set by the provider. Having these data has been useful for guiding treatment and education.

**Figure:**
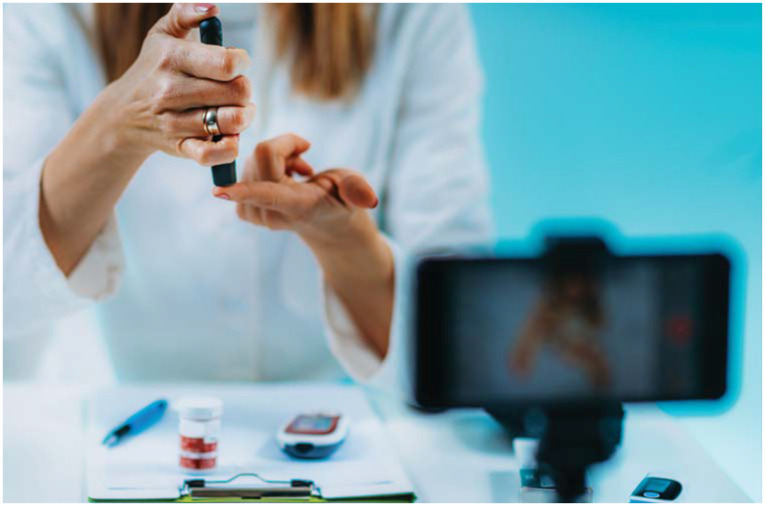


Another valuable tool has been prescribing personal continuous glucose monitors (CGMs) and having individuals download the data to a website where providers can view it. Prior to COVID-19, I did not know too much about using CGMs, so I enrolled in a Continuous Glucose Monitoring Certificate Program on the the Association of Diabetes Care and Education Specialists (ADCES) website. This certificate program, which is currently free to members, gave me the knowledge and confidence to help people with diabetes understand CGM data for diabetes self-management.

Telehealth also proved helpful for teaching people how to use a device and seeing exactly what they were eating.

## Conclusion

Although the COVID-19 pandemic has been a difficult time, as a result of it, I was forced to find new ways to reach out to people with diabetes. I discovered that a method of communication that has been around for over 100 years—telephone calls—could be quite effective for medication management and for providing diabetes education. Telehealth also proved helpful for teaching people how to use a device and seeing exactly what they were eating. Radio allowed me to reach out to our Spanish-speaking population. Having curbside A1Cs made this test more accessible to patients. Zoom presentations granted me the opportunity to offer diabetes education across state boundaries. Telemonitoring became a valuable tool, and registry reports enabled me to identify individuals who would most benefit from diabetes education.

Ultimately, the percentage of people we see having an A1C over 9 and those who had not had this test done within the year significantly decreased during the pandemic at our clinic compared to the previous years.

For that, I am grateful for these silver linings. ■
